# Changes in dietary carbon footprint over ten years relative to individual characteristics and food intake in the Västerbotten Intervention Programme

**DOI:** 10.1038/s41598-019-56924-8

**Published:** 2020-01-08

**Authors:** Therese Hjorth, Ena Huseinovic, Elinor Hallström, Anna Strid, Ingegerd Johansson, Bernt Lindahl, Ulf Sonesson, Anna Winkvist

**Affiliations:** 1 Department of Internal Medicine and Clinical Nutrition, the Sahlgrenska Academy, University of Gothenburg, Gothenburg, Sweden; 2 Department of Agrifood and Bioscience, RISE- Research Institutes of Sweden, Gothenburg, Sweden; 3 Department of Odontology, Umeå University, Umeå, Sweden; 4 Department of Public Health and Clinical Medicine, Section of Sustainable Health, Umeå University, Umeå, Sweden

**Keywords:** Environmental impact, Epidemiology, Risk factors

## Abstract

The objective was to examine 10-year changes in dietary carbon footprint relative to individual characteristics and food intake in the unique longitudinal Västerbotten Intervention Programme, Sweden. Here, 14 591 women and 13 347 men had been followed over time. Food intake was assessed via multiple two study visits 1996–2016, using a 64-item food frequency questionnaire. Greenhouse gas emissions (GHGE) related to food intake, expressed as kg carbon dioxide equivalents/1000 kcal and day, were estimated. Participants were classified into GHGE quintiles within sex and 10-year age group strata at both visits. Women and men changing from lowest to highest GHGE quintile exhibited highest body mass index within their quintiles at first visit, and the largest increase in intake of meat, minced meat, chicken, fish and butter and the largest decrease in intake of potatoes, rice and pasta. Women and men changing from highest to lowest GHGE quintile exhibited basically lowest rates of university degree and marriage and highest rates of smoking within their quintiles at first visit. Among these, both sexes reported the largest decrease in intake of meat, minced meat and milk, and the largest increase in intake of snacks and, for women, sweets. More research is needed on how to motivate dietary modifications to reduce climate impact and support public health.

## Introduction

Food production and consumption generate a large proportion of greenhouse gas emissions (GHGE) globally. Estimates show that agriculture, including deforestation, is responsible for approximately 24% of anthropogenic GHGE^1^, and that food production contributes with 19–29% of GHGE globally^2^. GHGE occur during all stages in the food system, from farming and its inputs to food distribution, consumption, and disposal of waste^3^. The main food-related emissions include methane, nitrous oxide, and carbon dioxide. Methane is produced by ruminants, during rice farming and manure management; nitrous oxide is produced from natural processes in the nitrogen cycle in agriculture and manure management; and carbon dioxide is produced from transports and during food processing using fossil fuels^4^. Food groups that produce high GHGE, i.e., have high dietary carbon footprint, include meat and dairy products. More specifically, livestock production contributes with 80% of agricultural GHGE globally^3^. However, there is a gradient in GHGE per kg meat between different meats, with beef and other ruminants yielding higher GHGE compared to pork and poultry. In contrast, many plant-based foods, such as legumes and root vegetables, yield relatively low GHGE compared to animal-based foods^5^. Hence, specific food choices among consumers have significant impact on total climate impact of diet and form a window for public health interventions to reduce global GHGE.

To achieve the global 2 °C climate target, emissions from agriculture as well as food production and consumption must be reduced, especially in affluent societies. Recent studies have shown that even if the effectiveness of productivity and agriculture increases, changes in food intake are still required to reach the climate target^6,7^. In addition, changes in intake of foods with high GHGE, such as meat, could bring health benefits as excessive intake of red and processed meat has been associated with cardiovascular disease, colorectal cancer, and type 2 diabetes^8,9^. In 2017 the average consumption of total meat in Sweden decreased for the first time following a steady upward trend since the end of the 1980s^10^. However, in international comparison Swedish meat consumption is high, both in relation to global and European average consumption^11,12^ levels. In addition, per capita intake levels of red meat in Sweden exceed the maximum 500 g per week recommended from a cancer perspective^13^. On the contrary average Swedish consumption of fruit, vegetables, legumes and whole grains are below recommended levels^14^. Thus, both environmental and health benefits could be achieved by changes in dietary intake^15^

In spite of the climate goals set globally as The Paris Agreement^16^ and in Sweden^17^ total GHGE have increased both nationally and globally over the last decades^18^. To reverse this trend and to achieve set goals large efforts are required, not least in the food sector. While scientific knowledge of how food products differ in climate impact is rather comprehensive^19^, research is more limited on how changes in dietary GHGE relate to individual characteristics and specific food choices in a longitudinal design, and the area is rapidly gaining increased scholarly interest^15,20,21^. Such information is valuable to identify target populations for public health interventions and develop effective policy instruments to reduce climate impact from diet. The aim of this study was to estimate 10-year changes in dietary carbon footprint in relation to individual characteristics and 10-year changes in food intake in a large population-based, longitudinal cohort in northern Sweden.

## Results

### Study subjects

**Figure 1:**
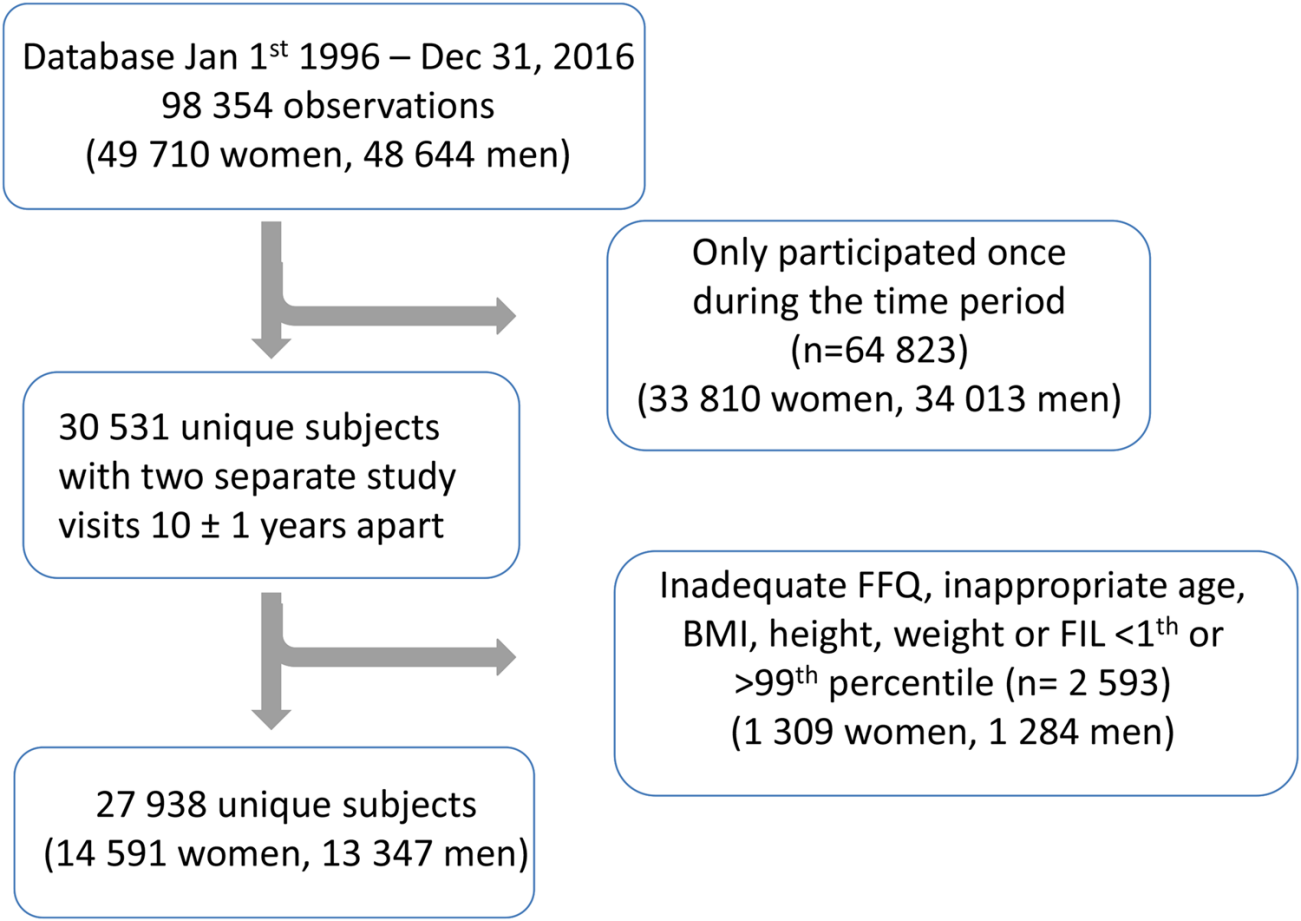
Sample selection to the study.

In total, 14 591 women and 13 347 men were included in the analyses (Fig. 1). Mean age at baseline was equal for women and men at the first and 10-year follow-up visits (Table 1). Over the 10 years, BMI increased by one unit and the proportion of smokers decreased for both women and men (Table 1).

**Table 1:** Background characteristics of participants in the Västerbotten Intervention Programme with 10-year follow-up data during 1996–2016 (n = 27 938).

Variable	Women (n = 14 591)		Men (n = 13 347)	
	Visit 1	Visit 2	Visit 1	Visit 2
Age^a^ (y)	44.9 (39.8, 50.3)	54.8 (49.7, 60.3)	44.9 (39.8, 50.3)	54.8 (49.7, 60.3)
BMI^a^ (kg/m^2^)	25.2 (19.9, 33.4)	26.2 (20.2, 35.2)	26.2 (21.5, 32.4)	27.2 (21.9, 34.3)
Energy intake^a^ (kcal/d)	1536 (1820, 1820)	1342 (1820, 1820)	2003 (1820, 1820)	1820 (1820, 1820)
Dietary carbon footprint^a^ (kg CO_2_e/1000 kcal and day)	3.03 (1.79, 4.66)	2.96 (1.67, 4.74)	3.88 (2.21, 6.16)	3.70 (2.02, 6.06)
**Marital status**^**b**^ **(%)**				
Married/cohabiting	85.0	81.5	81.6	80.5
Unmarried	6.2	6.7	11.5	11.0
Divorced/separated	7.8	9.3	6.7	7.8
Widow/widower	1.0	2.5	0.2	0.7
**Education**^**b**^ **(%)**				
Basic level (9 y)	11.5	10.6	13.3	13.2
High school	52.0	50.2	62.5	61.2
University	36.5	39.2	24.2	25.6
**Physical activity**^**b**^ **(%)**				
Inactive	16.4	16.0	17.8	17.2
Moderately inactive	31.6	26.3	30.4	26.5
Moderately active	27.8	26.8	29.3	29.3
Active	24.2	30.9	22.5	26.8
**Smoker**^**b**^ **(%)**				
Current	19.8	13.3	16.3	11.8
Former	31.5	35.7	30.6	33.0
Never	48.7	51.0	53.1	55.2
**Swedish snus (snuff)**^**b**^ **(%)**				
Current	8.7	8.3	13.5	25.1
Former	5.0	6.0	17.1	21.9
Never	86.3	85.7	51.4	53.0

^a^Mean (5, 95 percentile values). Adjustment for age at screening and screening year did not alter the values.

^b^The proportion missing values were for marital status 0.6% for women and men, respectively, at the first visit and 0.6% for women and 0.5% for men at the second visit; for education 0.5% for women and 0.3% for men at the first visit and 0.6% for women and 0.4% for men at the second visit; for physical activity index 0.1% for women and 0.2% for men at the first visit and 0.3% for women and men, respectively, at the second visit; for smoking 0.6% for women and 1.5% for men at the first visit and 1.0% for women and 1.6% for men at the second visit, and for Swedish snus 6.9% for women and 4.2% for men at the first visit and 2.6% for women and 1.7% for men at the second visit.

### Dietary carbon footprint (dietary GHGE)

**Figure 2:**
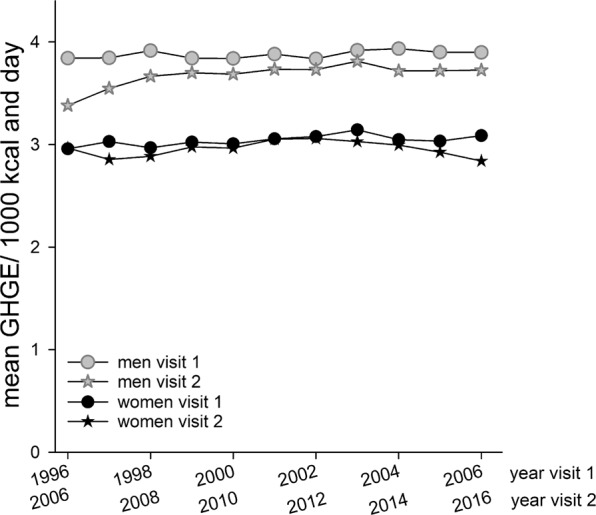
Dietary carbon footprint, expressed as mean greenhouse gas emissions (GHGE) per 1000 kcal and day, for men and women by year of study visit and standardized for age in the Västerbotten Intervention Programme.

Mean (95% CI) dietary carbon footprint for the 20-year study period (1996–2016) was 3.38 (3.37, 3.39) kg CO_2_e/1000 kcal and day, with values adjusted for sex, age and screening year. Mean dietary carbon footprints (95% CI) were slightly higher for the first visit [3.44 (3.43, 3.45)] than the follow-up visit [3.32 (3.30, 3.33)], but generally the dietary carbon footprint exhibited small changes over study years. Women had about 20% lower dietary carbon footprint than men for the same amount of calories **(**Fig. 2, Table 1). The estimated difference in dietary carbon footprint between the second and first study visit for a person ranged from a reduction of 11.2 to an increase of 10.9 kg CO_2_e/1000 kcal and day.

### Food intake and individual characteristics in relation to dietary carbon footprint at both visits

**Figure 3:**
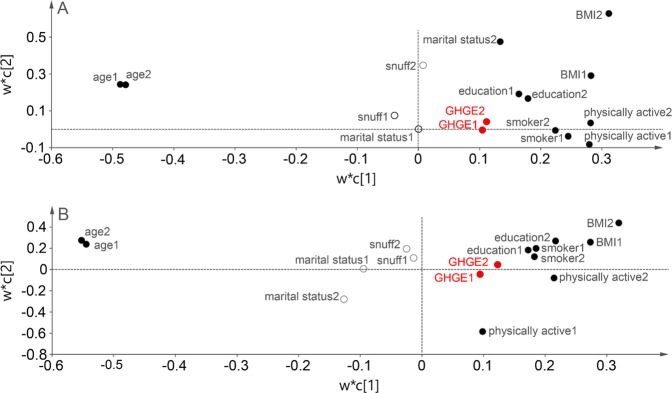
(**A,B)** Results from PLS modelling of individual characteristics influential to greenhouse gas emissions (GHGE) per 1000 kcal and day for women (panel A) and men (panel B) in the Västerbotten Intervention Programme. w*c [1] and w*c [2] indicate the weights for the different characteristics in relation to the outcome GHGE for the two most important components c [1] and c [2] created among the individual characteristics. Values related to study visit 1 are indicated with “1” and values related to study visit 2 with “2”. Filled circles indicate influential and open circles non-influential characteristics. Variables located close to or to the right of GHGE are associated with high levels and those to the left with low levels. BMI, body mass index; PLS, Partial Least Squares.

**Figure 4:**
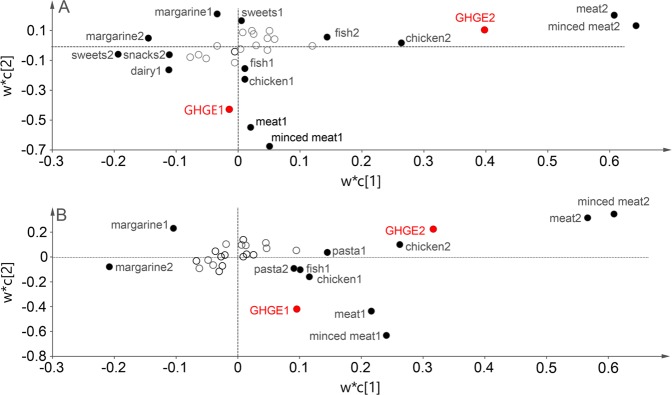
(**A,B**) Results from PLS modelling of foods influential to greenhouse gas emissions (GHGE) per 1000 kcal and day for women (panel A) and men (panel B) in the Västerbotten Intervention Programme. w*c [1] and w*c [2] indicate the weights for the different characteristics in relation to the outcome GHGE for the two most important components c [1] and c [2] created among the individual foods. Values related to study visit 1 are indicated with “1” and values related to study visit 2 with “2”. Filled circles indicate influential and open circles non-influential foods. Variables located close to or to the right of GHGE are associated with high levels and those to the left with low levels.

In Partial Least Square (PLS) modelling, at both visits, high BMI was most strongly associated with having the highest GHGE scores and old age with the lowest GHGE scores for both sexes with 2.3% and 2.6% of the GHGE variations explained (R^2^) by the model in women and men, respectively, and 2.2% and 2.4% of the variation predicted (Q^2^) according to the cross validation and with component 1 (c[1]) being statistically significant; Fig. 3A,B). Further, at both visits, intakes of meat and minced meat per 1000 kcal were most influential for having the highest GHGE scores, and intake of margarine for having the lowest GHGE scores for both sexes. Here, the R^2^-values were 26.5% and 23.2% for women and men, respectively, and the corresponding Q^2^-values 25.8% and 22.4%, respectively and both c [1] and c [2] being statistically significant; Fig. 4A,B). In addition, for women intake of sweets and snacks were most influential for having the lowest GHGE scores at the second visit, and sweets at the first visit.

### Food intake by quintile of dietary carbon footprint at study visits 1 and 2

In support of the results in Fig. 4, at both study visits and for both sexes, high GHGE (i.e., belonging to higher quintiles) was associated with higher median intakes of meat, minced meat, fish, butter, root vegetables, vegetables and pasta, and lower median intakes of chicken, margarine, rice, sweets and snacks per 1000 kcal and day (Table 2; see Methods section for details about each food group). Between study visits 1 and 2, there was a general trend for both sexes of increasing the consumption of meat, chicken, fish, butter, root vegetables and vegetables, and decreasing the consumption of margarine, potatoes, rice and pasta per 1000 kcal and day.

**Table 2:** Intake of 15 food groups by quintile of greenhouse gas emissions (GHGE) at visits 1 and 2 in the Västerbotten Intervention Programme^a.^

	N	GHGE	Meat	Minced meat	Chicken	Fish	Butter	Margarine	Milk products	Cheese
**Women**										
visit 1:Q1	2917	2.0 (0.3)	6.9 (3.2, 9.3)	8.4 (5.3, 10.9)	8.2 (5.3, 11.7)	9.9 (4.9, 15.3)	1.3 (0, 7.5)	8 (3.2, 15.7)	179 (96, 259)	8 (3.5, 13.9)
visit 1:Q2	2919	2.5 (0.2)	6.7 (5.3, 10.3)	8.3 (6.9, 12.9)	7.6 (6.0, 11.4)	10.6 (6.5, 15.3)	1.0 (0.0, 7.0)	7.4 (3.1, 15.2)	195 (127, 290)	9.7 (4.7, 13.1)
visit 1:Q3	2919	2.9 (0.2)	7.5 (5.1, 9.8)	8 (6.4, 14.4)	7.5 (5.6, 10.5)	10.4 (6.8, 14.9)	2.1 (0.0, 9.9)	7.0 (2.9, 14.4)	212 (138, 299)	9.3 (4.5, 12.6)
visit 1:Q4	2919	3.4 (0.2)	7.6 (4.9, 9.9)	10.7 (6.2, 16.3)	7.7 (5.2, 10.6)	10.7 (7.2, 14.8)	1.9 (0.0, 9.4)	6.7 (3.0, 13.4)	227 (144, 307)	9.0 (5, 12.5)
visit 1:Q5	2917	4.4 (0.7)	7.7 (5.6, 11.9)	13.4 (8.4, 22.3)	7.3 (5.2, 11.2)	11.2 (7.4, 15.8)	2.9 (0.0, 8.6)	6.1 (2.8, 11.9)	223 (150, 301)	8.4 (5.3, 15.5)
visit 2:Q1	2917	1.8 (0.3)	8.9 (5.8, 12.8)	9.9 (6.7, 15.1)	12.2 (8.4, 17.7)	16.4 (9.8, 24.4)	6.4 (1.3, 12.3)	2.9 (0, 7.9)	195 (111, 285)	7.8 (3.6, 12.9)
visit 2:Q2	2919	2.4 (0.2)	8.9 (6.2, 12.7)	10.4 (7.3, 16.5)	11.9 (8.1, 16.9)	16.3 (10.4, 23.5)	6.3 (1.3, 11.9)	2.9 (0, 7.5)	199 (119, 297)	8.3 (4.2, 12.4)
visit 2:Q3	2919	2.8 (0.2)	8.9 (6.1, 12.8)	10.2 (7.1, 16.3)	11.7 (8.1, 17.1)	15.9 (10.3, 23.2)	6.5 (1.6, 11.5)	2.8 (0, 7.1)	203 (133, 297)	8.3 (4.2, 12)
visit 2:Q4	2919	3.3 (0.2)	8.8 (6.2, 12.7)	10.8 (7.2, 17.9)	11.5 (7.9, 17.7)	16.4 (10.8, 23.4)	6.8 (1.8, 11.5)	2.6 (0, 7)	210 (140, 298)	8.3 (4.4, 12.1)
visit 2:Q5	2917	4.4 (0.8)	9.2 (6.3, 13.4)	12.2 (7.6, 20.6)	11.5 (7.9, 19.8)	16.2 (10.6, 24)	6.9 (2.2, 11.4)	2.5 (0, 6.8)	210 (141, 291)	7.8 (4.2, 11.4)
**Men**										
visit 1:Q1	2668	2.4 (0.4)	7.5 (5, 9.7)	8.2 (6.2, 12)	7.6 (5.1, 10.4)	8.8 (2.5, 13.9)	5.1 (0.1, 12.9)	8.9 (3.2, 17.6)	173 (101, 259)	6.4 (2.7, 11.6)
visit 1:Q2	2671	3.1 (0.2)	7.3 (5.3, 9.9)	9.5 (6, 12.1)	7.2 (5.2, 9.9)	9.1 (5.6, 14.1)	5.8 (0.6, 13.7)	8.8 (4.0, 16.3)	189 (122, 294)	7.1 (3.8, 11.2)
visit 1:Q3	2669	3.7 (0.2)	7.0 (5.5, 10.4)	9.1 (6.2, 13.6)	6.6 (5.0, 10.1)	9.3 (5.6, 13.5)	6 (0.8, 13.2)	8.6 (3.7, 15.7)	213 (129, 298)	7.7 (3.9, 11.2)
visit 1:Q4	2671	4.3 (0.3)	7.6 (5.3, 10.5)	10.3 (7, 19.2)	6.5 (4.8, 10.1)	9.6 (5.8, 13.9)	7.2 (1.1, 12.9)	7.6 (3.3, 13.9)	211 (128, 289)	7.6 (3.9, 10.7)
visit 1:Q5	2668	5.8 (1.1)	8.2 (5.7, 13.6)	15.1 (8.4, 21.7)	7.6 (4.6, 11.0)	9.7 (6.2, 14.3)	8.4 (1.5, 12.9)	6.6 (2.8, 13.1)	199 (125, 274)	7.2 (4.2, 12.4)
visit 2:Q1	2668	2.2 (0.3)	8.2 (5.6, 11.7)	9.4 (6.4, 13.7)	9.5 (6.4, 14.1)	12.3 (7.3, 19)	8.7 (1.9, 16.5)	4.5 (0.6, 10.7)	173 (91, 271)	6.4 (2.7, 11.5)
visit 2:Q2	2671	3.0 (0.2)	8.3 (5.8, 11.9)	9.5 (6.5, 14.6)	9.5 (6.7, 13.8)	12.5 (7.7, 18.2)	9.3 (2.7, 15.7)	4.5 (0.8, 10.4)	175 (105, 271)	7.1 (3.3, 11.3)
visit 2:Q3	2669	3.5 (0.2)	8.3 (5.7, 12)	9.8 (6.5, 15.6)	9.1 (6.3, 13.6)	12.5 (7.5, 18.2)	9.9 (3.4, 15.4)	4.3 (0.8, 9.6)	188 (115, 277)	7.1 (3.5, 11)
visit 2:Q4	2671	4.2 (0.3)	8.4 (5.8, 12.1)	10.1 (6.6, 17.3)	9.3 (6.2, 14)	12.6 (8, 18.5)	9.7 (3.6, 15)	4 (0.6, 9.4)	182 (112, 269)	7.3 (3.9, 11.1)
visit 2:Q5	2668	5.7 (1.1)	8.5 (5.9, 12.8)	11.3 (7.2, 19)	9.2 (6, 15.1)	12.7 (8.2, 18.7)	10.1 (4.3, 15)	3.8 (0.6, 8.5)	179 (107, 261)	7.2 (3.7, 10.9)
		**N**	**GHGE**	**Root vegetables**	**Vegetables**	**Potatoes**	**Rice**	**Pasta**	**Sweets**	**Snacks**
**Women**										
visit 1:Q1		2917	2.0 (0.3)	20.8 (7.2, 46)	41.9 (19.8, 77.4)	71.2 (46.8, 105.3)	18.3 (12.1, 28.5)	24.2 (14.9, 45.6)	2.9 (1.9, 6.2)	1.9 (0.1, 2.6)
visit 1:Q2		2919	2.5 (0.2)	22 (8, 44.5)	42.5 (20.9, 76.5)	71.1 (48.5, 103.4)	16.4 (11.2, 31.3)	26.2 (14.3, 44.3)	2.5 (1.7, 5.9)	1.6 (0.1, 2.5)
visit 1:Q3		2919	2.9 (0.2)	21.9 (8.3, 44)	42.6 (22.6, 73.2)	68.4 (45.8, 101)	15.1 (10.8, 31.8)	27.5 (13.7, 44.1)	2.3 (1.5, 5.4)	1.5 (0.1, 2.2)
visit 1:Q4		2919	3.4 (0.2)	23 (9.7, 44.3)	44 (23.8, 73.7)	67.8 (45.4, 98.2)	15.2 (10.7, 32.3)	27.0 (14.5, 42.2)	2.1 (1.5, 4.9)	1.4 (0.8, 2.1)
visit 1:Q5		2917	4.4 (0.7)	23.6 (10.5, 41.1)	43.4 (23.3, 74.8)	68.4 (45.1, 95.5)	15.1 (10.2, 30.7)	29.0 (15.4, 40.6)	1.9 (1.4, 4.3)	1.2 (0.8, 1.8)
visit 2:Q1		2917	1.8 (0.3)	26.7 (10, 55.8)	51.3 (25.4, 98.6)	50.4 (25.9, 81)	13.8 (9, 22.2)	15.3 (9.7, 28.6)	2.4 (1.6, 4.2)	1.7 (0.1, 2.6)
visit 2:Q2		2919	2.4 (0.2)	28.7 (12.2, 56.1)	54.4 (27.8, 98.4)	52.6 (28.9, 81)	13.4 (8.7, 20.6)	15.6 (9.7, 30.2)	2.4 (1.5, 4.5)	1.7 (0.1, 2.5)
visit 2:Q3		2919	2.8 (0.2)	28.6 (13.1, 52.5)	53.5 (28.8, 95)	52.3 (29.8, 81.7)	12.9 (8.4, 20.8)	15.5 (9.6, 30.6)	2.3 (1.5, 4.8)	1.6 (0.1, 2.4)
visit 2:Q4		2919	3.3 (0.2)	29.2 (13.6, 52.4)	54.1 (30.4, 95.3)	51.8 (29.9, 81.5)	13.1 (8.5, 23.2)	16.1 (9.6, 30.6)	2.2 (1.5, 4.7)	1.5 (0.1, 2.3)
visit 2:Q5		2917	4.4 (0.8)	29.6 (14.9, 51.7)	55.1 (30.8, 96.7)	55.6 (34.2, 85.3)	13 (8.2, 25.8)	18.3 (10.1, 32.8)	2 (1.3, 4.3)	1.4 (0.1, 2.2)
**Men**										
visit 1:Q1		2668	2.4 (0.4)	5.2 (2.2, 13.2)	14.5 (5, 30.3)	62.5 (38.5, 89.1)	15.1 (10.4, 22.5)	22.5 (13.8, 43.2)	2.3 (1.5, 3.7)	2.0 (0.1, 2.9)
visit 1:Q2		2671	3.1 (0.2)	5.2 (2.4, 14)	16.1 (5.4, 31.3)	61.0 (41.8, 88.8)	13.8 (9.3, 20.6)	22.5 (13.4, 41.2)	2.1 (1.3, 4.5)	1.8 (1.2, 2.7)
visit 1:Q3		2669	3.7 (0.2)	5.2 (2.4, 14.1)	16.0 (5.8, 31.6)	58.3 (41.9, 87.2)	12.8 (8.7, 24.2)	22.9 (13.2, 38.1)	1.9 (1.3, 4.3)	1.6 (1.2, 2.4)
visit 1:Q4		2671	4.3 (0.3)	6.5 (2.7, 15)	17.3 (7.4, 33.3)	57.3 (40.1, 83.9)	12.4 (8.9, 27.1)	26.6 (13.1, 37.9)	1.7 (1.2, 4.0)	1.4 (1.1, 2.2)
visit 1:Q5		2668	5.8 (1.1)	6.6 (2.5, 14.2)	17 (6.2, 31.9)	59.7 (40.5, 84.8)	12 (7.9, 25.4)	26.5 (13.7, 39.2)	1.7 (1.1, 3.6)	1.3 (0.9, 1.9)
visit 2:Q1		2668	2.2 (0.3)	6.7 (2.9, 18)	18.7 (6.2, 37)	49.1 (28.2, 74)	12.9 (8.5, 20.6)	15.5 (9.7, 28.1)	1.7 (1, 2.7)	1.8 (0.1, 2.8)
visit 2:Q2		2671	3.0 (0.2)	7.6 (3.2, 18.2)	20.3 (7.6, 39.4)	50 (30.2, 72.9)	12.6 (8.3, 19.2)	16.6 (10.4, 30.4)	1.6 (1.1, 2.7)	1.8 (0.1, 2.6)
visit 2:Q3		2669	3.5 (0.2)	8.3 (3.3, 18.2)	20.1 (8.3, 39.2)	48.9 (31.4, 72.5)	12.3 (8.1, 21.2)	16.8 (10, 31.2)	1.6 (1, 2.7)	1.7 (0.1, 2.6)
visit 2:Q4		2671	4.2 (0.3)	8.5 (3.3, 19.9)	21 (8.3, 39.9)	49.1 (30.5, 74.4)	11.7 (7.8, 22.3)	17.6 (9.9, 31.6)	1.5 (0.9, 2.9)	1.6 (0.1, 2.4)
visit 2:Q5		2668	5.7 (1.1)	9 (3.3, 18.6)	21.1 (8.5, 38.2)	51.2 (33.5, 76.4)	11.9 (7.8, 23.1)	18.7 (10.1, 32.3)	1.4 (0.9, 2.9)	1.5 (0.8, 2.3)

^a^GHGE-values are mean (SD) and expressed as kg CO_2_e/1000 kcal and day and adjusted for age. All other values are median (25 and 75 percentile values) and expressed as g/1000 kcal and day. Q, quintile.

### Change of dietary carbon footprint quintile over the study period

When dietary GHGE quintile positions were compared for visits 1 and 2, similar patterns with respect to individual characteristics and food intake were revealed for both sexes. Women and men who changed from the lowest quintiles (i.e., Q1) at visit 1 to Q5 at visit 2 (i.e., increased their dietary carbon footprint over the study period) had the highest BMI within their quintiles at visit 1 (Tables 3 and 4). Further, these women and men who increased their dietary carbon footprint over the study period reported the largest increase in consumption of meat, minced meat, chicken, fish and butter and the largest decrease in their consumption of potatoes, rice and pasta per 1000 kcal and day (Tables 5 and 6).

**Table 3:** Individual characteristics for women by quintile combinations for visits 1 and 2 (mean (SD) and %) in the Västerbotten Intervention Programme.

Quintile	N	GHGE	GHGE	BMI		University education, %		Married/cohabiting, %		Physically active, %		Present smoker, %	
group^a^		visit 1	visit 2	visit 1	10-year change, %	visit 1	visit 2	visit 1	visit 2	visit 1	visit 2	visit 1	visit 2
11	1220	1.89 (0.3)	1.78 (0.3)	25.0 (4.3)	3.9 (9.9)	32.8	35.7	79.5	75.8	20.1	28.8	23.0	16.0
12	743	1.98 (0.3)	2.39 (0.2)	24.7 (4.1)	4.8 (9.4)	35.1	38.1	82.9	80.1	24.1	30.2	22.7	14.0
13	488	2.01 (0.2)	2.81 (0.2)	24.9 (3.8)	5.2 (9.3)	37.5	39.4	83.0	81.5	19.1	29.7	19.0	12.0
14	296	2.03 (0.2)	3.27 (0.2)	25.3 (4.1)	5.1 (8.7)	36.3	39.2	81.3	79.4	19.6	28.0	22.8	16.8
15	170	2.02 (0.3)	4.32 (0.8)	25.6 (4.2)	3.7 (10.1)	32.1	36.3	80.1	77.4	21.3	34.7	22.2	12.2
21	761	2.50 (0.2)	1.86 (0.2)	25.0 (3.9)	4.4 (9.7)	30.3	32.5	82.7	76.5	23.8	29.6	24.0	15.7
22	759	2.52 (0.2)	2.40 (0.2)	25.0 (3.9)	3.7 (8.7)	34.4	35.9	85.4	82.7	24.8	29.6	19.6	13.4
23	623	2.53 (0.2)	2.83 (0.2)	24.9 (4.1)	5.0 (8.6)	37.7	40.6	85.3	81.3	23.6	31.0	20.2	14.5
24	479	2.54 (0.2)	3.29 (0.2)	25.1 (4.4)	5.2 (9.0)	37.4	39.9	83.6	83.2	23.2	30.2	22.2	13.6
25	297	2.54 (0.2)	4.29 (0.8)	25.3 (4.7)	5.0 (9.3)	41.4	44.1	85.5	83.3	23.6	27.8	19.7	12.5
31	466	2.90 (0.2)	1.88 (0.3)	25.3 (4.3)	4.1 (9.2)	33.7	36.1	83.3	77.4	22.0	27.1	25.9	15.0
32	657	2.91 (0.2)	2.41 (0.2)	24.8 (3.8)	4.9 (9.5)	34.1	37.2	87.8	82.9	22.1	28.8	20.8	14.5
33	692	2.91 (0.2)	2.82 (0.2)	25.2 (4.2)	4.4 (8.8)	37.1	39.6	86.7	83.6	22.7	29.3	20.0	13.7
34	655	2.93 (0.2)	3.33 (0.2)	24.8 (4.0)	4.4 (9.0)	38.5	41.1	87.9	87.2	22.5	32.2	18.3	12.4
35	449	2.94 (0.2)	4.26 (0.7)	25.5 (4.9)	4.9 (9.8)	40.7	42.8	86.1	82.6	22.7	32.4	15.9	10.8
41	321	3.38 (0.2)	1.92 (0.2)	25.5 (4.8)	3.9 (10.3)	25.1	29.3	81.8	73.8	27.4	25.7	19.7	13.5
42	482	3.37 (0.2)	2.42 (0.2)	24.9 (4.1)	4.6 (8.9)	34.4	37.8	86.0	83.9	23.9	29.6	20.9	14.1
43	651	3.38 (0.2)	2.83 (0.2)	25.0 (4.3)	4.5 (9.4)	35.2	39.3	88.9	82.7	29.2	33.6	16.8	12.5
44	748	3.39 (0.2)	3.32 (0.2)	25.2 (4.2)	4.4 (9.1)	39.1	42.5	86.7	85.6	25.3	31.4	15.3	10.8
45	717	3.40 (0.2)	4.30 (0.7)	25.4 (4.5)	4.1 (8.6)	40.3	43.5	87.5	85.3	25.4	33.2	15.3	11.3
51	149	4.20 (0.5)	1.93 (0.2)	25.7 (5.5)	3.7 (12.3)	36.7	38.8	78.5	70.9	30.4	30.4	23.6	17.1
52	278	4.33 (0.8)	2.42 (0.2)	25.4 (4.8)	5.6 (10.3)	38.5	39.7	83.1	81.2	20.9	32.7	22.7	14.4
53	465	4.25 (0.6)	2.84 (0.2)	24.9 (4.1)	4.2 (8.9)	35.8	39.2	87.1	79.0	27.2	31.3	18.9	12.8
54	741	4.32 (0.7)	3.34 (0.2)	25.5 (4.8)	4.1 (8.8)	39.1	41.7	87.4	83.4	28.6	32.6	17.6	13.2
55	1284	4.51 (0.8)	4.60 (0.9)	26.0 (4.7)	4.6 (10.0)	41.7	43.9	86.8	83.8	28.5	35.6	17.3	10.5

^a^Groups formed based on quintile positions for greenhouse gas emissions per 1000 kcal and day at visits 1 and 2. Group 11 refers to being categorized into quintile 1 at both visits; ranking done within sex and 10 year age strata. GHGE, greenhouse gas emission.

**Table 4:** Individual characteristics for men by quintile combinations for visits 1 and 2 (mean (SD) and %) in the Västerbotten Intervention Programme.

Quintile	N	GHGE	GHGE	BMI		University education, %		Married/cohabiting, %		Physically active, %		Present smoker, %	
group^a^		visit 1	visit 1	visit 1	10-year change, %	visit 1	visit 2	visit 1	visit 2	visit 1	visit 2	visit 1	visit 2
11	1202	2.34 (0.4)	2.16 (0.4)	26.0 (3.4)	4.1 (7.7)	18.8	20.5	75.4	74.5	19.8	24.1	17.5	13.0
12	663	2.47 (0.3)	2.93 (0.2)	26.4 (3.6)	3.7 (7.1)	21.1	23.9	78.5	79.1	19.8	25.9	14.9	10.7
13	422	2.50 (0.3)	3.46 (0.2)	26.6 (3.7)	3.7 (8.0)	22.0	25.4	77.2	78.1	20.9	24.5	15.1	12.0
14	249	2.57 (0.3)	4.14 (0.3)	26.2 (3.2)	4.1 (7.0)	25.4	27.3	76.6	77.7	25.5	32.3	17.5	13.7
15	132	2.52 (0.3)	5.55 (1.1)	27.3 (4.0)	5.3 (9.3)	23.1	24.4	70.8	79.2	17.4	29.5	18.3	11.5
21	661	3.13 (0.2)	2.28 (0.3)	26.2 (3.3)	3.9 (7.5)	17.7	18.0	77.8	76.9	21.2	21.0	19.3	13.2
22	747	3.14 (0.2)	2.96 (0.2)	26.2 (3.4)	3.5 (7.1)	22.5	23.4	81.0	82.6	21.1	26.4	14.7	10.9
23	583	3.15 (0.2)	3.49 (0.2)	26.1 (3.6)	4.6 (7.2)	24.7	25.8	83.9	82.8	19.6	24.2	13.8	9.3
24	439	3.16 (0.2)	4.10 (0.3)	26.0 (3.4)	4.5 (7.9)	27.8	27.2	84.5	84.4	19.4	26.8	16.9	13.3
25	241	3.19 (0.2)	5.41 (0.8)	26.7 (3.4)	4.1 (7.3)	24.2	23.3	76.6	77.8	22.0	30.3	13.9	9.2
31	446	3.67 (0.2)	2.29 (0.3)	26.1 (3.0)	3.5 (7.4)	22.2	22.3	80.3	80.0	22.5	24.0	17.3	13.1
32	570	3.68 (0.2)	2.96 (0.2)	25.9 (3.1)	3.9 (6.5)	21.1	22.2	84.6	83.8	21.3	26.4	17.9	11.8
33	664	3.69 (0.2)	3.49 (0.2)	25.7 (3.2)	3.4 (7.0)	27.0	28.9	84.9	82.5	22.3	27.3	14.1	10.4
34	569	3.72 (0.3)	4.16 (0.3)	26.2 (3.3)	3.8 (6.8)	25.1	25.9	84.1	82.5	23.9	28.0	14.4	8.9
35	420	3.72 (0.2)	5.46 (0.9)	26.5 (3.5)	4.0 (7.6)	29.0	32.3	79.0	81.5	21.2	28.7	17.9	14.4
41	229	4.34 (0.3)	2.30 (0.3)	26.4 (4.1)	3.3 (7.6)	17.5	19.7	76.4	70.6	25.4	24.1	20.3	15.0
42	440	4.31 (0.3)	2.97 (0.2)	26.1 (3.7)	3.8 (8.1)	23.6	24.5	83.7	79.8	23.4	25.1	15.9	11.5
43	591	4.33 (0.3)	3.49 (0.2)	26.1 (3.1)	4.1 (7.2)	27.1	28.1	84.2	82.3	22.8	27.8	16.9	14.6
44	728	4.37 (0.3)	4.17 (0.3)	26.1 (3.6)	3.4 (7.5)	27.1	28.5	84.8	81.7	26.8	29.1	15.7	12.1
45	683	4.37 (0.3)	5.52 (1.0)	26.4 (3.5)	3.6 (6.8)	28.5	30.6	84.9	83.7	21.5	31.3	13.6	9.4
51	130	5.75 (1.4)	2.37 (0.3)	26.8 (3.8)	3.1 (7.3)	19.2	20.0	77.7	71.5	34.6	25.6	25.0	17.5
52	251	5.52 (0.7)	3.00 (0.2)	26.5 (3.3)	3.7 (7.7)	22.8	24.3	86.1	79.1	19.7	23.6	17.8	16.2
53	409	5.58 (0.9)	3.52 (0.2)	26.0 (3.4)	2.8 (7.0)	26.0	28.8	86.1	84.0	22.7	28.9	16.8	12.1
54	686	5.62 (0.9)	4.17 (0.3)	26.2 (3.6)	3.7 (7.1)	26.0	27.2	85.1	81.9	25.1	25.1	16.3	10.0
55	1192	5.99 (1.3)	5.89 (1.3)	26.5 (3.9)	4.2 (7.6)	28.3	30.2	82.0	81.7	27.1	31.0	16.4	11.7

^a^Groups formed based on quintile positions for greenhouse gas emission per 1000 kcal and day at visits 1 and 2. Group 11 refers to being categorized into quintile 1 at both visits; ranking done within sex and 10 year age strata. GHGE, greenhouse gas emission.

**Table 5:** Food intakes among women in quintile combinations for visits 1 and 2. Intakes are median intakes in g/1000 kcal and day at visit 1 and the change between visits 1 and 2 in median intakes in g/1000 kcal (continues on next page) in the Västerbotten Intervention Programme.

Quintile	N	Meat		Minced meat		Chicken		Fish		Butter		Margarine		Milk products		Cheese	
group^a^		visit 1	visit diff	visit 1	visit diff	visit 1	visit diff	visit 1	visit diff	visit 1	visit diff	visit 1	visit diff	visit 1	visit diff	visit 1	visit diff
11	1220	7	0.32	8.4	0.55	8.5	3.23	9.8	5	1.4	1	7.9	−2.9	181	0.7	8.0	−0.32
12	743	7	2.18	8.3	1.65	8.2	4.2	9.8	6.3	1.5	1.7	8.1	−3.6	178	11.4	8.1	−0.39
13	488	6.8	3.38	8.5	3.48	7.9	4.47	9.2	6.8	1	2.5	8	−4	185	22.1	8.3	−0.03
14	296	6.8	3.20	8.1	4.17	8.1	4.45	11.3	7.7	2	2.5	7.9	−3.2	175	33.8	7.7	−0.03
15	170	6.8	7.67	8.6	6.98	8.2	7.5	11.1	8.5	0.9	2.5	7.2	−4.1	169	34.3	6.7	−0.91
21	761	7	0.14	8.4	0.2	7.9	3.63	10.6	4.9	0.9	1.9	7.5	−2.7	200	−24.4	9.2	−0.59
22	759	6.7	1.30	8.3	1.23	7.6	3.35	10.5	4.9	0.9	1.4	7.5	−3.1	192	3.9	9.9	−0.58
23	623	6.6	1.49	8.3	1.1	7.4	4.18	10.6	4.9	1.3	1.8	7.3	−3.5	198	13.4	10.2	−0.72
24	479	6.5	2.58	8.2	3.51	7.3	4.2	10.7	5.7	1.4	1.7	7.4	−3.6	195	21.7	9.7	−0.76
25	297	6.6	6.55	8.3	8.08	8.2	6.88	11	8.5	0.8	3	7.2	−4.8	180	4.8	8.3	0.05
31	466	7.9	0.08	9.1	−0.91	7.7	3.42	10.7	4.4	1.2	1.2	7.1	−2.6	225	−31.2	9.5	−0.74
32	657	7.1	1.22	8	0.56	7.6	3.4	10.5	4.8	2.5	1.1	7.3	−2.7	220	−4.3	9.7	−0.23
33	692	7.4	1.22	7.8	1.13	7.1	3.62	10.5	4.9	1.8	1.4	6.8	−2.8	219	2.5	9.4	−0.67
34	655	7.1	1.83	7.8	1.97	7.4	3.86	10.2	5.5	3	1.2	6.6	−2.9	206	2.6	9.2	−0.51
35	449	8.1	3.52	8	5.86	7.8	5.12	10.4	7	1.9	2.4	7.1	−3.9	190	−2.6	8.8	−0.44
41	321	7.9	0.05	11.3	−1.67	7.8	2.99	11.1	4.8	1.5	1.2	7.6	−1.9	244	−47.4	8.9	−0.49
42	482	8	0.55	10.7	−1.27	7.8	3.33	10.9	4.5	1.4	2.2	7.1	−2.8	231	−11.6	9.2	−0.57
43	651	7.4	1.22	10.5	0.57	7.6	3.11	10.5	4.9	2.5	2	6.7	−2.3	234	−9.2	9.1	−0.19
44	748	7.3	1.35	10.4	1.34	7.5	3.86	10.5	4.9	2.7	1.4	6.5	−3.2	224	2.8	9.1	−0.37
45	717	7.5	2.71	10.8	4.25	7.7	4.69	10.7	6.5	1.7	2.2	6.3	−3.1	210	2.7	8.7	−0.55
51	149	9.2	−3.43	12	−3.13	8.1	2.85	12	3.7	3.8	2.2	5.4	−0.7	222	−24.8	7.9	−1.84
52	278	8.2	−0.21	13.6	−3.62	7.4	3.16	10.7	4.3	3.6	2.1	6.2	−1.9	215	−29.7	8.5	−1.34
53	465	7.9	−0.04	13.4	−3.38	7.1	3.55	11.3	3.9	3.5	1.4	5.2	−1.6	226	−8.1	8.7	−0.49
54	741	7.3	0.69	13.3	−1.71	7.2	3.02	11.1	4.4	3.1	1.9	6.8	−2.5	230	5.4	8.4	−0.72
55	1284	7.7	2.17	13.5	2.04	7.4	4.32	11.3	5.5	2.2	1.9	6.5	−2.7	221	−0.9	8.3	−0.83
Quintile	N	Root vegetables		Vegetables		Potatoes		Rice		Pasta		Sweets		Snacks			
group^a^		visit 1	visit diff	visit 1	visit diff	visit 1	visit diff	visit 1	visit diff	visit 1	visit diff	visit 1	visit diff	visit 1	visit diff		
11	1220	19.4	3.2	42.0	6.8	69.9	−14.7	18.6	−2.0	23.9	−5.1	2.9	−0.03	2.0	0.01		
12	743	22.6	2.5	42.1	8.3	72.8	−19.9	18.0	−3.6	23.9	−6.4	2.9	−0.26	1.9	0.00		
13	488	18.6	3.1	41.1	7.3	77.1	−20.8	17.6	−4.7	24.8	−8.0	2.8	−0.4	1.8	−0.01		
14	296	25.2	1.0	42.4	8.9	66.3	−21.9	18.0	−4.9	25.3	−9.6	3.0	−0.78	1.8	−0.03		
15	170	25.6	0.9	41.5	5.1	68.5	−26.0	19.7	−7.8	26.7	−12.6	2.9	−1.05	1.8	−0.12		
21	761	21.9	6.1	42.8	11.1	69.2	−15.2	16.3	−1.0	24.9	−4.9	2.5	0.12	1.7	0.04		
22	759	19.8	3.8	39.6	8.8	69.8	−13.3	16.0	−1.9	23.8	−5.9	2.5	0.01	1.6	0.02		
23	623	22.2	3.9	44.5	8.2	71.9	−16.0	16.1	−2.7	25.2	−5.5	2.6	−0.19	1.7	0.00		
24	479	24.6	1.2	44.6	5.9	72.5	−16.2	16.9	−4.2	27.9	−6.5	2.5	−0.5	1.6	−0.05		
25	297	24.8	2.9	43.7	10.9	75.2	−25.5	17.7	−7.2	31.8	−9.6	2.5	−0.56	1.6	−0.07		
31	466	21.4	1.9	42.7	8.8	65.7	−11.5	14.7	−0.1	25.6	−5.2	2.3	0.38	1.5	0.34		
32	657	20.6	5.8	42.4	9.0	65.5	−12.9	15.3	−1.8	25.3	−5.1	2.3	0.15	1.5	0.05		
33	692	23.2	4.5	44.2	10.0	68.7	−13.0	15.3	−2.3	28.0	−6.3	2.3	0.02	1.5	0.02		
34	655	21.3	4.8	41.5	8.1	68.3	−12.3	15.2	−2.7	28.5	−7.6	2.3	−0.15	1.4	0.00		
35	449	23.7	5.3	42.3	11.4	73.6	−22.8	15.3	−4.2	28.4	−7.4	2.2	−0.39	1.5	−0.05		
41	321	20.7	3.2	39.2	7.3	68.1	−15.2	16.1	−2.2	26.0	−5.5	2.2	0.55	1.4	0.43		
42	482	21.3	3.7	43.7	9.0	67.0	−11.6	14.6	−2.0	25.1	−5.6	2.1	0.43	1.4	0.14		
43	651	22.1	5.4	45.2	7.9	67.2	−13.6	15.0	−1.9	25.0	−4.8	2.2	0.17	1.3	0.09		
44	748	22.5	4.3	43.2	9.5	68.4	−11.2	14.8	−1.8	27.9	−6.1	2.1	−0.02	1.3	0.02		
45	717	26.5	3.8	47.3	9.5	67.7	−17.8	15.6	−3.5	29.9	−9.1	2.1	−0.27	1.3	−0.01		
51	149	21.3	4.3	41.4	9.5	61.6	−7.2	15.4	−1.2	24.6	−5.4	1.6	0.8	1.2	0.77		
52	278	21.8	4.9	36.3	9.4	67.9	−8.5	13.8	−0.9	27.4	−6.7	2.0	0.25	1.2	0.47		
53	465	21.7	3.2	40.7	6.4	67.1	−10.4	14.1	−1.5	26.9	−6.7	1.9	0.31	1.3	0.21		
54	741	22.5	5.2	42.3	9.4	69.1	−9.1	14.2	−1.1	29.0	−5.3	2.0	0.16	1.2	0.12		
55	1284	25.9	4.6	46.4	8.4	69.6	−11.7	17.8	−2.0	29.9	−6.8	1.9	−0.11	1.2	0.01		

^a^Groups formed based on quintile positions for greenhouse gas emission equivalents per 1000 kcal and day at visits 1 and 2. Group 11 refers to being categorized into quintile 1 at both visits; ranking done within sex and 10 year age strata.

**Table 6:** Food intakes among men in quintile combinations for visits 1 and 2. Intakes are median intakes in g/1000 kcal and day at visit 1 and the change between visits 1 and 2 in median intakes in g/1000 kcal (continues on next page) in the Västerbotten Intervention Programme.

Quintile	N	Meat		Minced meat		Chicken		Fish		Butter		Margarine		Milk products		Cheese	
group^a^		visit 1	visit diff	visit 1	visit diff	visit 1	visit diff	visit 1	visit diff	visit 1	visit diff	visit 1	visit diff	visit 1	visit diff	visit 1	visit diff
11	1202	7.5	0.26	8.4	0.14	7.7	1.35	8.8	2.6	5.2	1.1	8.9	−2.1	175	−10.9	6.5	−0.05
12	663	7.4	1.10	8.0	0.53	7.3	1.81	8.4	3.8	5.3	1.2	8.8	−3.1	176	4.8	6.1	−0.01
13	422	7.5	1.47	8.0	1.77	7.7	2.58	9.0	3.7	5.1	1.4	9.1	−3.6	169	18.0	7.1	−0.06
14	249	7.6	3.10	7.8	3.72	7.6	2.22	9.6	4.4	4.9	1.6	9.9	−4.2	168	4.2	6.2	0.55
15	132	7.8	7.02	8.4	8.88	8.0	4.12	8.8	6.0	3.4	5.3	9.0	−3.6	164	−8.5	6.0	0.68
21	661	7.6	0.02	9.7	−0.56	7.0	2.05	8.6	2.9	6.3	1.0	8.0	−1.8	203	−26	7.1	−0.13
22	747	7.3	0.59	9.4	0.35	7.3	1.79	8.7	3.0	6.1	0.8	8.8	−2.4	193	−10	7.2	0.12
23	583	7.2	0.66	9.6	0.43	7.2	1.57	9.7	3.2	5.6	1.7	9.3	−2.8	196	−11.9	7.4	0.00
24	439	7.2	1.96	9.0	2.04	7.3	2.62	9.6	3.6	4.7	1.9	9.2	−3.9	176	−6.6	6.8	0.05
25	241	7.4	4.33	9.8	5.79	7.1	3.05	9.9	2.9	6.0	3.1	9.5	−4.0	161	7.5	7.1	−0.4.0
31	446	7.1	−0.09	9.2	−1.01	6.7	1.96	9.3	1.8	6.6	0.9	8.4	−1.4	194	−38.4	7.6	−0.66
32	570	7.0	0.74	9.0	0.23	6.5	2.07	9.1	3.7	5.3	1.1	9.7	−2.8	213	−9.1	8.0	−0.05
33	664	6.8	0.64	9.1	0.53	6.5	1.69	9.2	2.4	6.5	1.0	7.7	−1.8	226	−13.4	7.3	0.00
34	569	6.8	0.80	9.0	0.73	6.5	2.28	9.4	3.3	6.2	1.0	8.6	−2.6	218	−8.5	8.2	−0.24
35	420	7.1	3.38	9.1	4.0	6.7	3.02	9.8	3.7	5.3	2.8	8.5	−3.9	201	−6.7	7.3	−0.16
41	229	7.0	−0.73	10.6	−2.66	7.1	2.65	9.3	2.8	9.2	0.0	7.0	0.0	217	−21.0	7.2	0.00
42	440	7.8	0.12	11.1	−1.76	6.4	2.44	8.9	2.8	7.0	1.4	6.9	−1.7	211	−23.1	7.6	−0.51
43	591	7.2	0.33	10.5	−1.0	6.6	2.04	9.5	2.0	6.7	1.0	8.1	−2.1	217	−4.3	7.7	0.17
44	728	7.3	0.92	10.0	0.14	6.3	1.53	9.7	2.9	8.1	0.7	7.6	−2.2	224	−13.0	7.5	0.04
45	683	8.0	1.87	9.5	2.11	6.5	2.77	10.0	2.9	6.5	1.2	7.8	−3.2	196	−9.6	7.9	−0.29
51	130	8.6	−1.11	14.6	−3.88	8.3	0.25	9.6	2.2	8.7	0.9	6.2	−0.1	204	−40.2	6.5	−0.07
52	251	8.5	−0.82	14.9	−4.46	7.5	1.32	9.0	3.0	8.4	1.1	7.6	−1.5	207	−23.4	7.4	−0.48
53	409	8.0	−0.30	14.9	−4.23	7.5	1.76	9.3	1.8	8.7	1.3	7.2	−1.4	202	−13.7	7.0	−0.09
54	686	8.1	−0.26	15.1	−2.74	7.4	1.30	9.7	2.0	8.4	1.2	6.5	−1.5	203	−5.3	7.5	−0.10
55	1192	8.3	0.95	15.4	−0.27	7.6	1.72	10.0	2.4	8.3	1.1	6.3	−2.1	191	−5.7	7.2	−0.26
**Quintile**	**N**	**Root vegetables**		**Vegetables**		**Potatoes**		**Rice**		**Pasta**		**Sweets**		**Snacks**			
**group**^**a**^		**visit 1**	**visit diff**	**visit 1**	**visit diff**	**visit 1**	**visit diff**	**visit 1**	**visit diff**	**visit 1**	**visit diff**	**visit 1**	**visit diff**	**visit 1**	**visit diff**		
**11**	**1202**	5.1	0.9	13.6	1.5	60.8	−8.7	14.9	−0.6	22.4	−4.9	2.3	−0.3	2.0	0.01		
12	663	5.1	0.8	15.4	1.4	63.6	−10.7	15.0	−1.8	21.2	−5	2.4	−0.61	2.0	−0.02		
13	422	5.2	0.8	15.9	2	61.6	−12.5	15.8	−3.5	24.2	−6.9	2.3	−0.74	1.9	−0.08		
14	249	4.9	1.1	14.4	1.5	65.9	−22.6	15.0	−3.5	23.9	−7.5	2.2	−0.71	2.0	−0.14		
15	132	6.9	−0.1	16.1	1	71.9	−20.8	16.4	−4.3	20.4	−6.4	2.4	−1.11	1.9	−0.30		
21	661	4.7	1	14.7	1.8	61.4	−9.5	13.6	0.1	20.9	−2.9	2.1	−0.21	1.8	0.03		
22	747	4.8	0.9	15.8	1.8	59.9	−8.8	13.8	−0.5	22.1	−2.8	2.1	−0.21	1.8	0.02		
23	583	5.7	0.7	17.9	1.8	60.9	−12	13.4	−0.7	21.9	−4.4	2.1	−0.34	1.7	−0.02		
24	439	5.9	0.7	16.4	1.9	64.2	−9.7	13.7	−1.9	24.6	−4.6	2.1	−0.47	1.7	−0.02		
25	241	5.6	1.4	16.6	2	59.3	−12.5	14.5	−4.9	28.8	−8.1	2.0	−0.69	1.8	−0.18		
31	446	4.7	1.3	14.7	1.2	58.3	−5.7	12.5	1.2	23.1	−2.7	1.8	0.02	1.5	0.23		
32	570	5.2	1	17.2	2.4	55.6	−9.4	12.4	0	21.5	−3.6	1.8	−0.15	1.6	0.10		
33	664	5.5	1.3	16.9	1.7	57.0	−7.6	13.0	−0.7	22.7	−3.4	1.9	−0.25	1.6	0.02		
34	569	5.2	1.1	15.8	3	58.4	−8.8	12.7	−0.8	22.6	−3.5	2.0	−0.58	1.6	−0.02		
35	420	5.3	1.8	15.3	4.6	62.6	−17.1	13.7	−2.5	27.3	−6.3	1.9	−0.66	1.5	−0.06		
41	229	5.3	1.1	16.2	0.2	55.0	−8.1	12.6	0.1	26.4	−3.5	1.6	0.02	1.4	0.35		
42	440	5.4	1	17.5	1	58.2	−5.6	12.1	0.2	23.5	−2.7	1.7	−0.01	1.5	0.32		
43	591	6.8	1.3	16.9	2.2	54.2	−7	12.1	−0.1	27.0	−3.8	1.8	−0.23	1.4	0.11		
44	728	6.5	1.1	17.4	2.1	60.5	−9	12.6	−1	25.8	−3.5	1.7	−0.35	1.4	0.01		
45	683	7.4	1.1	18.1	1.9	60.2	−10	12.7	−2.4	28.6	−5.6	1.8	−0.54	1.5	−0.08		
51	130	5.6	1.6	12.5	3.7	60.1	−8.2	13.1	0.4	26.6	−2.9	1.5	−0.15	1.2	0.56		
52	251	5.4	1.4	16.1	4.9	57.1	−3.4	11.9	−0.3	25.0	−6	1.7	−0.03	1.4	0.42		
53	409	6.8	0.9	17.5	2.6	62.3	−9.7	11.4	1	25.9	−4.3	1.6	−0.06	1.3	0.13		
54	686	6.2	1	17.0	1.6	59.0	−5.6	11.9	0.4	24.7	−2.7	1.7	−0.29	1.3	0.05		
55	1192	7.2	1.3	17.0	2.3	58.8	−6.2	12.2	−1.3	27.6	−3.7	1.6	−0.35	1.4	0.00		

^a^Groups formed based on quintile positions for greenhouse gas emission equivalents per 1000 kcal and day at visits 1 and 2. Group 11 refers to being categorized into quintile 1 at both visits; ranking done within sex and 10 year age strata.

Both women and men who changed from the highest GHGE quintiles at visit 1 (i.e., Q5) to Q1 at visit 2 (i.e., decreased their dietary carbon footprint over the study period) had within their quintiles at visit 1 basically the lowest rates of university degree and marriage and highest rates of smoking (Tables 3 and 4). Further, these women and men who decreased their dietary carbon footprint over the study period reported the largest increase of consumption of snacks (and women also of sweets) and among the largest decrease of consumption of meat, minced meat and milk per 1000 kcal and day (Tables 5 and 6). The observed dietary changes were the same for men and women except for a reported increased intake of cheese among women who reduced their dietary carbon footprint, which was not observed for men.

## Discussion

The results of this unique longitudinal population-based study on changes in food intake and changes in GHGE over time show that women and men moving from lower to highest dietary carbon footprint quintile over a ten-year period were characterized by having higher BMI initially. Increased dietary carbon footprint over the study period was moreover associated with an increased reported intake of meat, minced meat, chicken, fish and butter and a decreased intake of potatoes, rice and pasta. Women and men who reduced their dietary carbon footprint over the study period exhibited lower rates of university degree and marriage and higher rates of smoking initially. Reduced dietary carbon footprint was for both sexes associated with a decreased reported intake of meat, minced meat and milk, and an increased intake of snacks (and sweets for women). At both visits, high BMI was most strongly associated with the highest dietary carbon footprint per 1000 calories and day, and old age with the lowest dietary carbon footprint per 1000 calories and day. Foods associated with the highest dietary carbon footprint scores were meat and minced meat, whereas margarine, sweets and snacks were associated with the lowest dietary carbon footprint scores. In sum, within this large population-based sample with repeated measurements from Northern Sweden changes in dietary carbon footprint were related to individual characteristics as well as to changes in food choices.

Our findings are in line with previous studies reporting that individuals with high dietary carbon footprint have higher intake of meat and dairy products^22,23^. Since foods from plant-based origin have relatively lower GHGE, a change towards a vegetarian or a vegan diet may reduce dietary carbon footprint^24–26^. An adoption of a vegetarian or vegan diet may also have health benefits^27^. However, exclusion of meat does not necessarily result in a decreased GHGE as the sum of such dietary modifications depends on what foods are chosen to replace the meat. For example, prepared pork has a GHGE of about 10 kg CO_2_e/kg, while the corresponding value for e.g., halloumi cheese is about 17 kg CO_2_e/kg^28^. Hence, well-informed, specific and effective changes in food choices are required to achieve a reduction in climate impact of diet.

In our study, reduced dietary carbon footprint over time was associated with both more healthy and unhealthy food choices. In line with previous studies^25,28–30^, our results suggest that increased consumption of unhealthy choices such as sweets and snacks correlates with lower dietary carbon footprint. This finding and the call for reduced intake of animal-based foods have raised concerns regarding the nutritional adequacy of diets mainly designed to reduce the dietary carbon footprint. Several studies have examined the link between healthy, nutritious, and climate-friendly diets with varying results^28,31,32^. A study in a French population found that GHGE can be reduced by 30% without any major changes in nutritional intake (by for example replacing beef with pork), whereas a larger reduction in GHGE may result in impaired nutritional quality^33^. However, the major challenge in achieving this reduction is how to motivate individuals to decrease their meat intake. According to research conducted in the Netherlands, motivation to change towards a vegetarian diet per se is low; nevertheless, a majority of the population reported that they were willing to decrease their meat intake^34^. Furthermore, a Swedish study recently examined the nutritional quality of a diet when meat consumption was decreased by 50% and replaced with grain legumes. The results showed that such dietary changes could improve nutritional quality while concurrently decrease dietary carbon footprint by up to 20%^22^. Thus, messages to reduce and replace meat consumption could be a fruitful alternative to solely suggesting an exclusion of these food groups.

An intriguing finding of this study is the result that men and women who decreased their dietary carbon footprint exhibited lower rates of university degree initially. A possible explanation may be that higher educational level, and thereby a higher income, enables more frequent consumption of meat, fish, and cheese, i.e. more expensive foods with higher GHGE. This hypothesis is supported by results from a national food survey in Sweden showing that individuals with median or above median income more often consume animal-based products compared to individuals with below median income^14^. However, other studies have not found any association between dietary carbon footprint and educational level^30^. Like previous studies^30^, our results show that dietary carbon footprint was higher for men than women also for the same amount of calories, indicating men as an important target group for policy instruments aiming to reduce climate impact from diets. In addition, our study showed that women and men who reduced their dietary carbon footprint were less often married and more often smokers initially. More studies are needed to show whether these relationships are general also in other populations and to understand underlying factors, to exclude the possibility that other personal characteristics confound the relationships found.

Furthermore, we used energy-adjusted GHGE values to examine changes in dietary carbon footprint so that diet quality, rather than amount of food consumed, would be captured in GHGE. At each study visit, high BMI was most strongly associated with the highest dietary carbon footprint per 1000 calories and day. Also, women and men moving from lower to highest dietary carbon footprint quintile over the study period had the highest BMI within their quintile at study start. This indicates that these individuals both consumed larger volumes of food at the first study visit to maintain their larger body (likely associated with higher total GHGE), and also had the largest increase in GHGE per calorie consumed during the study period. In future research, GHGE should preferably be linked to a functional unit such as health-associated nutrient profile of the food and not only to its energy content, as it is the combined benefit of low GHGE and high nutrient profile that is of importance to consumers interested in planetary as well as human health. Examples of research in this field is presented by Hallström *et al*.^35^.

Another interesting finding from our study is that lower GHGE was associated with older age. This finding is in line with results from a Spanish study that examined the relation between food patterns and sociodemographic factors and found that young men were more likely to consume a classic western diet high in meat and fat^36^. Also, other research report that older women consume less animal-based products and more vegetables compared to younger women^37^. In the contrary, a recent study found that younger women and men were more likely to consume a diet low in GHGE^30^. These aspects call for further research on age-related trends in dietary carbon footprint.

The Västerbotten Intervention Programme is a large population-based cohort where previous reports have shown little evidence of selection bias^38^. The present study benefits from having recent life cycle analyses data for all food items analysed in the applied Food Frequency Questionnaire. Although GHGE values based on life cycle analysis include some uncertainty^39,40^, efforts have been made to increase the reliability of results. One of the challenges of estimating dietary carbon footprints of complete diets is the need to collect data from different life cycle studies that may vary in methodological choices. In order to harmonize data, GHGE values for all food items in the present analysis used the same system boundaries and were consistently re-calculated into prepared form^41^. Furthermore, the life cycle data used were calculated to be representative for average Swedish food consumption, considering variations in GHGE due to differences in origin and production methods. The choice of in part using weighted averages, where GHGE values of each food item reflect the mean emissions caused by foods from different production methods, mean that variation in GHGE might differ between individuals with similar diet. This is not critical for the results presented but needs to be kept in mind in the broader discussion on sustainable food systems and the role of production systems and diets.

In order to reveal patterns in dietary carbon footprint, individual characteristics and dietary intake over time, solely descriptive results have been presented. We believe that our large population-based data set on these variables offers a unique possibility to evaluate complex patterns and that our descriptive results represent the best way to share our results; statistical tests would run the risk of mass significance and many comparisons would be significant only because of our large sample size. Further, results where individuals exhibiting extreme results on a variable at a first measurement move closer to mean values for the group at a second time point likely harbours the phenomenon *regression towards the mean*. Part of the changes in GHGE and diet intake over time that we describe for individuals in the lowest or highest quintiles of GHGE at study start may be explained by this phenomenon, but our results demonstrate changes beyond this explanation in that these individuals even surpass the mean values of their group. Hence, the major part of noted changes are likely real.

As for the limitations, the FFQ was primarily designed to examine risk factors for cardiometabolic diseases^42^; hence, it was not designed specifically to capture foods with varying GHGE. However, national statistics and national time trends were used to calculate proportions of different meats consumed in Sweden over the study period and corresponding numbers were used to specify food items in the FFQ. National statistics were also used to estimate shares of e.g., domestic and imported vegetables and fruits. Still, no data are available on how well these national statistics apply to the examined area in northern Sweden. Second, participants reported lower energy intake at the second study visit, likely reflecting increased underreporting over time as both BMI and reported physical activity increased concurrently. The anticipated underreporting was handled by the energy standardization of dietary carbon footprint and food intake. Also, changes and trends in intake of specific food groups relevant from a climate-perspective (e.g., meat, dairy products, fish, rice etc.) may still be valid as these foods are not included in the “target food groups” for selective underreporting (e.g., sugars, sweets, snacks, sodas)^43^. Finally, ideally, more environmental aspects than climate impact should be considered when examining food intake, but this was not possible in this study due to lack of data on e.g., eutrophication and eco-toxicity as well as land and water use.

## Conclusion

This unique longitudinal study shows that changes in dietary carbon footprint over ten years were associated with individual characteristics at study start and changes in food choices, which were both healthy and unhealthy. We found that individuals with the greatest increase in dietary carbon footprint per calorie over ten years had higher BMI initially, whereas individuals with the greatest decrease in dietary carbon footprint had lower educational level, were less often married and smoked more initially. For the same amount of calories, women had about 20% lower dietary carbon footprint than men. More research is needed to explore how to motivate dietary modifications to support public health and reduce climate impact of diet.

## Methods

### Study design and subjects

The Västerbotten Intervention Programme (VIP) is an ongoing population-based prospective study initiated in 1985 in Västerbotten county in northern Sweden. The project started in the municipality of Norsjö in 1985 and by 1991 covered the entire county^42^. The intervention initially combined a population-based strategy encompassing the entire population, with an individual strategy where inhabitants were invited to screening and health counselling meetings at their primary health care centre. The former part included public information meetings, activities in non-governmental organizations and invitations to study groups and physical activities, but these activities ceased over time. The individual strategy however is still on-going. Here, each year inhabitants who turn 40, 50 or 60 years are invited to their local health care centre for a health screening. Before 1996, 30-year olds were also invited, and still are in some communities^42^. The health screening includes measurement of e.g., height, weight, and blood pressure. Participants also answer a comprehensive questionnaire that, besides diet, covers socioeconomic and psychosocial conditions, such as working conditions, physical activity, and alcohol and tobacco use^42^. During 1985–2016, approximately 120 000 individuals have participated in the study, of which more than 30 500 individuals have participated twice^44^. Written and informed consent was obtained from all participants and the study adhered to the Helsinki Declaration. The Research Ethics Committee at Umeå University approved the original study in 1984 (Dnr 2013/332/31) and the Regional Ethics Examination Board in Gothenburg approved the current study in 2019 (Dnr 2019–00986).

### Dietary assessment

At the study visit, participants answer a validated semi-quantitative food frequency questionnaire (FFQ) that covers the whole diet over the previous 12 months^45^. During 1985–1996, the FFQ included 84 food items but from 1996, a shorter (64-item) version was introduced^42^. Four colour photographs of increasing portion sizes are used to estimate intake of staple foods, vegetables, and meat/fish. The FFQ has nine consumption frequencies, ranging from “none” to “four times or more per day” (https://www.umu.se/en/biobank-research-unit/).

### Sample selection

In this study, participants who had completed the shorter FFQ version and who had two repeated study visits within 10 ± 1 years were included. In total, 30 531 individuals aged 29–65 years with two separate study visits ten years apart any time between 1996 and 2016 were identified. In line with other publications from the VIP study, additional exclusions were made for individuals who had missed indicating the portion sizes, individuals with > 10% of the FFQ questions missing, and individuals with a food intake level (reported energy intake divided by estimated basal metabolic rate) <1^st^ percentile or >99^th^ percentile. Furthermore, individuals were excluded if data on height and/or weight were missing. Body mass index (BMI) was calculated as weight/height^2^. Individuals with BMI <15 kg/m^2^, weight <35 kg and, height <130 cm or >210 cm were excluded resulting in 27 938 individuals available for analysis (Fig. 1).

### Estimation of GHGE

To estimate dietary GHGE, the 64 food items from the FFQ were categorised into ten main food groups including 54 sub-groups of food. Each subgroup of food was linked to specific GHGE from life cycle assessment studies, expressed in carbon dioxide equivalents (CO_2_e) per kg food product (see Supplementary Table 1). To capture variation in GHGE within a sub-group of foods, emission values were in part based on weighted averages reflecting differences in GHGE due to food type (e.g. type of meat) and production method (e.g. greenhouse vs. open field production) (see Supplementary Table 1 for further details). For example, GHGE for red meat was calculated as a weighted average of beef, pork, mutton and game based on national consumption statistics (www.jordbruksverket.se), taking in consideration changes in consumption over the 20-year study period (1996–2016).

As different GHGE have different global warming potential (GWP), weighting factors are used to create the common unit CO_2_e per kg food product. The GWP factors used in life cycle analyses may vary due to differences in choice of calculation method and assumptions^46^. Here, the GWP factors used are based on a 100-year time horizon. For plant-based foods, except rice, GWP factors from the 4^th^ assessment report by the Intergovernmental Panel on Climate Change (IPCC)^47^, were used, i.e., 1 for carbon dioxide, 25 for methane, and 298 for nitrous oxide. However, for animal-based foods and rice where methane emissions in the production are most significant the updated GWP factor for methane, 34, from the 5^th^ assessment report (IPCC) was used^18^.

The system boundaries used were primary production up to and including the retail phase. Emissions after retail phase such as consumer transportation, storing, cooking, and waste management were not included, nor were emissions related to land-use change. Emissions of CO_2_e were calculated per kg edible food product, e.g., meat without bone. If the life cycle assessment studies indicated food items in raw form, re-calculations were made to the prepared form^48^. For all foods, emissions from food waste along the studied life cycle was included, waste fractions along the life cycle were calculated based on estimates in studies^6,49^ (see Supplementary Table S1 for further details).

The majority of the life cycle assessment data used matched the selected system boundaries, i.e. included GHGE from primary production up to and including retail phase. When this was not the case, standard emissions were added for different stages in the food system according to the method described by Sjörs *et al*.^4^. For composite dishes in the FFQ, 1–3 ingredients were chosen to represent the dish. The GHGE of the dish was estimated by either the proportion of the ingredients that had the greatest importance to the weight of the dish or the climate impact. All recipes for composite dishes came from the national Swedish food composition database^48^.

### Changes in food intake and dietary carbon footprint over study period

To aid interpretability of the results, fifteen food groups were created from the FFQ based on GHGE of the specific foods (Table 7). Food groups with relatively high GHGE, i.e., ≥ 1.2 kg CO_2_e/kg, such as meat, fish, and dairy products, as well as food groups with relatively low GHGE, i.e., < 1.2 kg CO_2_e/kg, such as root vegetables, vegetables, and pasta, were chosen for analysis. Food intake was expressed as grams /1000 kcal of reported total intake per day, and dietary carbon footprint as kg CO_2_e/1000 kcal and day, to adjust for differences in reported energy intake and thus reflect diet quality rather than intake size.

**Table 7:** Food items included in the food groups used for analyses of changes in food intake over the 10-year follow-up period.

Food group	Food items
Red meat	Whole meat and dishes with whole meat (weighted for consumption proportions of beef, pork, mutton, game meat and time trends in these proportions)
Minced meat dishes	Minced meat dishes (beef)
Chicken	Chicken, hen
Fish	All fish types
Butter^a^	Butter blends, butter for bread spread, butter used in cooking
Margarine^a^	Margarine as bread spread, margarine used for cooking
Milk products^a^	Non-fermented milk, fermented milk, cream
Cheese^a^	Cheese of high and low fat
Root vegetables	Carrots, red beet, rutabaga, parsnip
Vegetables	Tomatoes, cucumber, salad, spinach, kale, broccoli
Potatoes	Boiled potatoes, fried potatoes, French fries
Rice	All kinds of rice
Pasta	All kinds of pasta
Sweets	Milk chocolate, dark chocolate, foam sweets, jelly sweets
Snacks	Chips, popcorn, peanuts

^a^All fat contents are incorporated.

Changes in food intake over the study period were calculated as intake at study visit 2 minus intake at study visit 1 (g/day). Participants were classified into quintile groups based on GHGE within sex and 10-year age group strata at first and second study visits (Q1 to Q5, with Q1 representing the lowest and Q5 the highest values). Quintiles at both study visits were inspected for individuals remaining in equivalent quintiles at both study visits or switching quintile position between study visits.

### Non-dietary variables

Physical activity level was measured using the Cambridge Index for Physical Activity, which is a validated index based on two questions related to physical activity level at work and leisure time^50^. Participants were categorised into inactive, moderately inactive, moderately active, and active. Smoking was categorised into current, former, and never; education level was categorised into basic level, high school, and university; and marital status was categorised into unmarried, married/cohabitant, divorced/separated, and widow/widower.

### Statistical analysis

Descriptive results are presented as means (SD, standard deviation) for numerical normally distributed variables, as medians (25^th^ and 75^th^ percentile) for non-normally distributed numerical variables and as % for categorical variables. Values presented as means were adjusted for individual characteristics as specified in each table using Generalized Linear Model (GLM). Quintiles of GHGE at first and second visit were constructed within age and sex specific strata. Partial Least Square (PLS) modelling was selected as the multivariate regression method to evaluate the association patterns between GHGE and individual characteristics as well as food intakes in sex-separate multivariate models with GHGE as dependent variables. Partial Least Squares modelling was used since it identifies directions in an X-swarm that characterize X well and are related to Y, allows a moderate skewness and covariation among X variables, and creates a few new variables containing most of the information for problem solving and displaying. The analyses were performed using Simca P + (version 15.0, Umetrics, Sartorius Stedim Biotech, Umeå, Sweden). The software autoscales and transforms the variables as appropriate. The explanatory (R2) as well as the predictive (Q2) power of the models were used to evaluate their goodness of fit. For Q2, the Simca P + software performs a K-fold cross-validation where 1/7^th^ of data are systematically kept out when fitting the model and predicted from the remaining data. All analyses except PLS were performed in IBM SPSS Statistics, version 25 (Armonk, NY: IBM Corp).

## Supplementary information

Supplementary Information

## Data Availability

The datasets generated and analyzed during the current study are not publicly available due to Swedish law, but are available from the corresponding author on reasonable request.

## References

[1] Smith, P. M. B. *et al*. Climate Change 2014. (Cambridge, United Kingdom and New York, NY, USA, 2014).

[2] Vermeulen SJ, Campbell BM, Ingram JS (2012). Climate change and food systems. Annual Review of Environment and Resources.

[3] Friel S (2009). Public health benefits of strategies to reduce greenhouse-gas emissions: food and agriculture. Lancet.

[4] Sjors C (2016). Diet-related greenhouse gas emissions assessed by a food frequency questionnaire and validated using 7-day weighed food records. Environ Health.

[5] Williams, A. G., Audsley, E. & Sandars, D. L. *Determining the environmental burdens and resource use in the production of agricultural and horticultural commodities*. (Cranfield University and Defra, Bedford, 2006).

[6] Bryngelsson D, Wirsenius S, Hedenus F, Sonesson U (2016). How can the EU climate targets be met? A combined analysis of technological and demand-side changes in food and agriculture. Food Policy.

[7] Springmann Marco, Clark Michael, Mason-D’Croz Daniel, Wiebe Keith, Bodirsky Benjamin Leon, Lassaletta Luis, de Vries Wim, Vermeulen Sonja J., Herrero Mario, Carlson Kimberly M., Jonell Malin, Troell Max, DeClerck Fabrice, Gordon Line J., Zurayk Rami, Scarborough Peter, Rayner Mike, Loken Brent, Fanzo Jess, Godfray H. Charles J., Tilman David, Rockström Johan, Willett Walter (2018). Options for keeping the food system within environmental limits. Nature.

[8] Farchi S, De Sario M, Lapucci E, Davoli M, Michelozzi P (2017). Meat consumption reduction in Italian regions: Health co-benefits and decreases in GHG emissions. PLoS One.

[9] Richi EB (2015). Health risks associated with meat consumption: a review of epidemiological studies. Int. J. Vitam. Nutr. Res..

[10] Board of Agriculture. Tydlig utveckling - vi äter mindre kött och mer svenskt (Strong development- we are eating less meat and more of Swedish origin) [In Swedish], [cited 2019-10-31], http://www.jordbruksverket.se/omjordbruksverket/pressochmedia/nyheter/nyheter2018/tydligutvecklingviatermindrekottochmersvenskt.5.42a946c0161df8b7b8f1958c.html (2018).

[11] Organisation for Economic Co-operation and Development (OECD). *Meat Consumption*, [cited 2019-10-31], https://data.oecd.org/agroutput/meat-consumption.htm (2018).

[12] Board of Agriculture. Livsmedelskonsumtion och innehåll (food consumption and food contents) [In Swedish], [cited 2019-10-31], http://www.jordbruksverket.se/webdav/files/SJV/Amnesomraden/Statistik,%20fakta/Livsmedel/JO44SM1801/JO44SM1801_tabeller4.htm (2019).

[13] Nordic Council of Ministers. *Nordic Nutrition Recommendations 2012: Integrating nutrition and physical activity*. 5:e ed edn, (Nordic Council of Ministers, Copenhagen, 2014).

[14] The Swedish National Food Administration. Riksmaten – vuxna 2010–11 - Livsmedels- och näringsintag bland vuxna i Sverige (Food and nutrition intake among adults in Sweden) [In Swedish]. (Livsmedelsverket, Uppsala, 2012).

[15] Davis KF (2016). Meeting future food demand with current agricultural resources. Global Environmental Change.

[16] United Nations. *The Paris Agreement* [cited 2019-12-03], https://unfccc.int/process-and-meetings/the-paris-agreement/the-paris-agreement (2019).

[17] Swedish Environmental Protection Agency. *Reduced Climate Impact*, [cited 2019-10-31], http://www.swedishepa.se/Environmental-objectives-and-cooperation/Swedens-environmental-objectives/The-national-environmental-objectives/Reduced-Climate-Impact/ (2019).

[18] Pachauri, R. K. *et al*. *Climate change 2014: synthesis report. Contribution of Working Groups I, II and III to the fifth assessment report of the Intergovernmental Panel on Climate Change*. (IPCC, 2014).

[19] Clune S, Crossin E, Verghese K (2017). Systematic review of greenhouse gas emissions for different fresh food categories. J. Clean Prod..

[20] Gephart JA (2016). The environmental cost of subsistence: optimizing diets to minimize footprints. Sci. Total Environ..

[21] Strid, A. *et al*. Climate impact from diet in relation to background and sociodemographic characteristics in the Västerbotten Intervention Programme. *Publ. Hlth. Nutr*. 1–10 (2019). 10.1017/S1368980019002131 PMC10260555 31566152

[22] Röös, E. *et al*. Less meat, more legumes: prospects and challenges in the transition toward sustainable diets in Sweden. *Renewable Agriculture and Food Systems*, 1–14, 10.1017/S1742170518000443.

[23] Gonzalez-Garcia S, Esteve-Llorens X, Moreira MT, Feijoo G (2018). Carbon footprint and nutritional quality of different human dietary choices. Sci. Total Environ..

[24] Masset G, Soler LG, Vieux F, Darmon N (2014). Identifying sustainable foods: the relationship between environmental impact, nutritional quality, and prices of foods representative of the French diet. J. Acad. Nutr. Diet..

[25] Scarborough P (2014). Dietary greenhouse gas emissions of meat-eaters, fish-eaters, vegetarians and vegans in the UK. Clim. Change.

[26] Hallström E, Carlsson-Kanyama A, Börjesson P (2015). Environmental impact of dietary change: a systematic review. J. Clean Prod..

[27] Key TJ, Appleby PN, Rosell MS (2006). Health effects of vegetarian and vegan diets. Proc. Nutr. Soc..

[28] Sjors C, Hedenus F, Sjolander A, Tillander A, Balter K (2017). Adherence to dietary recommendations for Swedish adults across categories of greenhouse gas emissions from food. Publ. Hlth Nutr..

[29] Vieux F, Soler L-G, Touazi D, Darmon N (2013). High nutritional quality is not associated with low greenhouse gas emissions in self-selected diets of French adults. Am. J. Clin. Nutr..

[30] Rose D, Heller MC, Willits-Smith AM, Meyer RJ (2019). Carbon footprint of self-selected US diets: nutritional, demographic, and behavioral correlates. Am. J. Clin. Nutr..

[31] Macdiarmid JI (2012). Sustainable diets for the future: can we contribute to reducing greenhouse gas emissions by eating a healthy diet?. Am. J. Clin. Nutr..

[32] Joyce A, Hallett J, Hannelly T, Carey G (2014). The impact of nutritional choices on global warming and policy implications: examining the link between dietary choices and greenhouse gas emissions. Energy and Emission Control Technologies.

[33] Perignon M (2016). How low can dietary greenhouse gas emissions be reduced without impairing nutritional adequacy, affordability and acceptability of the diet? A modelling study to guide sustainable food choices. Publ. Hlth Nutr..

[34] van de Kamp ME (2018). Healthy diets with reduced environmental impact? - The greenhouse gas emissions of various diets adhering to the Dutch food based dietary guidelines. Food Res. Int..

[35] Hallström E, Davis J, Woodhouse A, Sonesson U (2018). Using dietary quality scores to assess sustainability of food products and human diets: a systematic review. Ecological Indicators.

[36] Sanchez-Villegas A, Delgado-Rodriguez M, Martinez-Gonzalez MA, De Irala-Estevez J (2003). Gender, age, socio-demographic and lifestyle factors associated with major dietary patterns in the Spanish Project SUN (Seguimiento Universidad de Navarra). Eur. J. Clin. Nutr..

[37] Mishra G, Ball K, Arbuckle J, Crawford D (2002). Dietary patterns of Australian adults and their association with socioeconomic status: results from the 1995 National Nutrition Survey. Eur. J. Clin. Nutr..

[38] Weinehall L, Westman G, Janlert U, Wall S (1998). Reduction of selection bias in primary prevention of cardiovascular disease through involvement of primary health care. Scand. J. Prim. Health Care.

[39] Röös, E. *Analysing the carbon footprint of food. Insights for consumer communication*, (Swedish University of Agricultural Sciences, Uppsala) (2013).

[40] Notarnicola B (2017). The role of life cycle assessment in supporting sustainable agri-food systems: A review of the challenges. J. Clean Prod..

[41] Heller MC, Keoleian GA, Willett WC (2013). Toward a life cycle-based, diet-level framework for food environmental impact and nutritional quality assessment: a critical review. Environ. Sci. Technol..

[42] Norberg Margareta, Wall Stig, Boman Kurt, Weinehall Lars (2010). The Västerbotten Intervention Programme: background, design and implications. Global Health Action.

[43] Lafay L (2000). Does energy intake underreporting involve all kinds of food or only specific food items? Results from the Fleurbaix Laventie Ville Sante (FLVS) study. Int. J. Obes..

[44] Umeå universitet. *Swedish National Data Service SND. Northern Sweden Diet Database (NSDD)*, https://snd.gu.se/en/catalogue/study/ext0131. (2017).

[45] Johansson I (2002). Validation and calibration of food-frequency questionnaire measurements in the Northern Sweden Health and Disease cohort. Publ. Hlth Nutr..

[46] IPCC. *Climate Change 2013 – The Physical Science Basis: Working Group I Contribution to the Fifth Assessment Report of the Intergovernmental Panel on Climate Change* (ed Intergovernmental Panel on Climate Change) 659–740 (Cambridge University Press, 2014).

[47] IPCC, 2007. *Climate Change 2007: Synthesis Report. Contribution of Working Groups I, II and III to the Fourth Assessment Report of the Intergovernmental Panel on Climate Change*. [Core Writing Team, Pachauri, R.K. and Reisinger, A., eds] (Cambridge, Cambridge Univ. Press, 2007).

[48] The Swedish National Food Administration. Livsmedelsdatabasen (The Swedish food composition database) [In Swedish], [cited 2019-10-31] http://www7.slv.se/SokNaringsinnehall (2018).

[49] Sjörs, C. *Näringsintag och utsläpp av växthusgaser från svenska matvanor ur ett epidemiologiskt perspektiv (Nutritional intake and greenhouse gas emissions from Swedish eating habits from an epidemiological perspective [in Swedish]* (2017).

[50] Peters T (2012). Validity of a short questionnaire to assess physical activity in 10 European countries. Eur. J. Epidemiol..

